# GFinisher: a new strategy to refine and finish bacterial genome assemblies

**DOI:** 10.1038/srep34963

**Published:** 2016-10-10

**Authors:** Dieval Guizelini, Roberto T. Raittz, Leonardo M. Cruz, Emanuel M. Souza, Maria B. R. Steffens, Fabio O. Pedrosa

**Affiliations:** 1 Department of Biochemistry and Molecular Biology, Federal University of Parana (UFPR), Curitiba, PR, Brazil; 2 Graduate Program in Bioinformatics, Sector of Professional and Technological Education, Federal University of Parana (UFPR), Curitiba, PR, Brazil

## Abstract

Despite the development in DNA sequencing technology, improving the number and the length of reads, the process of reconstruction of complete genome sequences, the so called genome assembly, is still complex. Only 13% of the prokaryotic genome sequencing projects have been completed. Draft genome sequences deposited in public databases are fragmented in contigs and may lack the full gene complement. The aim of the present work is to identify assembly errors and improve the assembly process of bacterial genomes. The biological patterns observed in genomic sequences and the application of a priori information can allow the identification of misassembled regions, and the reorganization and improvement of the overall *de novo* genome assembly. GFinisher starts generating a Fuzzy GC skew graphs for each contig in an assembly and follows breaking down the contigs in critical points in order to reassemble and close them using jFGap. This has been successfully applied to dataset from 96 genome assemblies, decreasing the number of contigs by up to 86%. GFinisher can easily optimize assemblies of prokaryotic draft genomes and can be used to improve the assembly programs based on nucleotide sequence patterns in the genome. The software and source code are available at http://gfinisher.sourceforge.net/.

After twenty years of the publication of the first bacterial genomes, only 13% of the prokaryotic genome sequencing projects in public databases is completely finished and the remaining deposited draft genome sequences have an average of 190 contigs^1^. The task of assembly the complete sequence of prokaryotic genomes using a shotgun approach and the short reads dataset provided by DNA sequencers and remains a challenge for Bioinformatists^2^. The taxonomic classification, the identification of genes and the prediction of the metabolic pathways, and also the amplification of regions of biotechnological interest may be difficult in draft genomes containing a large number of contigs^3^. All genome assemblers are based on the assumption of sequence overlap among read sequences in a dataset to extend the sequences into contigs and reconstruct the original DNA sequence. However, the presence of repetitive regions, and errors introduced by the sequencing process can make the process unfeasible or very difficult computationally leading to genome misassemblies. The identification of repeat regions is hampered due to errors in bases calling and in the depth of coverage variation^4^ and they are the main causes of the exponential explosion in the number of nodes in, for instance, the de-Bruijn graph^5^. The failure to identify repeated sequences can lead to the assembly of compressed genomes as in the case of *Rhodobacter sphaeroides*. In the originally deposited genome 7, 5% of the genome was missing^4^,^5^,^6^. Even when an assembler correctly gauges the number of repeated copies, misassemblies still occur due to repeat facilitated inversions^4^.

Few metrics are universally used particularly in announcements of “draft” genomes. There are software that evaluate different aspects of the assembly. COMPASS provides information regarding coverage, validity, multiplicity and parsimony^7^; PLANTAGORA evaluates: performance, installation and representativeness^8^. QUAST^9^ incorporated and introduced new metrics. PLANTAGORA and QUAST proposed ways to identify assembly failures in contigs and to evaluate the representativeness and fragmentation of genes. However, most of these metrics are dependent on the existence of a closely related genome as reference.

The GAGE (Genome Assembly Gold-standard Evaluations) study was designed to provide a snapshot of how the latest genome assemblers compare on a sample of large-scale next-generation sequencing projects^10^. GAGE-B is an evolution of GAGE and conducted a comparison of the 8 main free assemblers utilizing 12 genome sequences of 8 organisms, and made available all pertaining data^11^.

The genome assembly programs are based on diverse assembly programs are based on diverse computational methods, which seek to find the best combination of sequence reads and to organize them in reliable arrangement representing the real sequence of the genome. However, these methods do not use biological information and bias such as the GC Skew to guide the assembly. This pattern recognition methodology is only used applied in the annotation process, for example, in gene prediction as implemented in Glimmer^12^ and GeneMarkS^13^ programs.

Lobry^14^ identified genomic compartmentalization of base frequencies and the equifrequency between A and T or between C and G. These biases may be determined by a GC skew, which measures the ratio between the number of guanines and the number of cytosine [(G − C)/(G + C)] along one strand of DNA molecule. This bias has been related to the cell replication process, with the predominance of base G in the leading strand of the DNA molecule being replicated and the origin and terminus of replication correspond to the minimum and the maximum points in the curve^15^,^16^. Systematic GC skew analyses of more than 400 published bacterial chromosome sequences revealed that rearrangements are rare^17^. COLLYN *et al*.^18^ describes chromosomal inversions in assembly of *Idiomarina loihiensis* L2TR resulting in inversion of trend of cumulative skew curves locally disrupting the symmetrical inverted V-shape.

In this work we present a new bioinformatics approach, based on GC skew to help finish prokaryotic genome sequences. The GFinisher pipeline implements algorithms to identify misassemblies, reorganize and reassemble bacterial genome. GFinisher requires two or more genome assemblies produced by different assemblers and a reference genome. The main steps of G-Finisher involve a) ordering the contigs of the target genomes using a reference genome of a closely related species, b) identification of potential contig misassemblies by the Fuzzy GC Skew newly developed algorithm and c) close scaffold gaps using the jFGap tool. Any of these steps can be run in sequence or independently.

## Results

**Figure 1:**
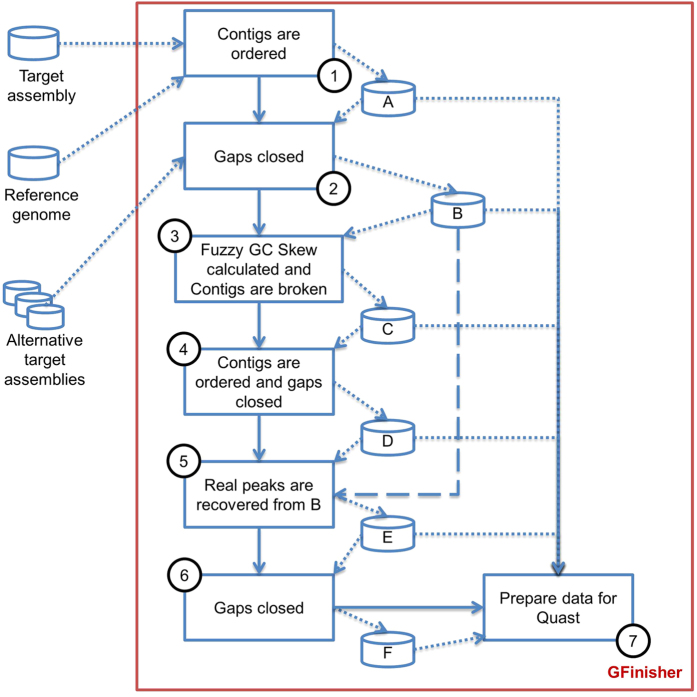
GFinisher workflows. A target assembly, some alternative assemblies and a reference genome sequence are inputs for the program. Numbered boxes represent the seven main steps in the analysis. A–F represents intermediate assemblies generated during the analysis. Solid lines show the process flows and dotted lines show the assembly flow. In step 5, intermediate assemblies B and D are compared to recover the true critical points (dashed line).

In this work we present the GFinisher, a new strategy to refine and finalize bacterial genome assembly. The main parameters and requirements of GFinisher are a target genome assembly, a set of alternative assemblies of the target genome, a phylogenetically close or species specific reference genome and a computational pathway to save the results (Fig. 1).

### GFinisher operating strategy

The program starts by ordering the contigs (Fig. 1-step 1) of the target assembly based on mapping of contigs to the reference genome composing new scaffolds of the target assembly (assembly A). In the next step the jFGap searches contigs in alternative assemblies produced by other assemblers that fill in gaps of Assembly A (Fig. 1-step 2), producing assembly B. In the third stage (Fig. 1-step 3) the Fuzzy GC Skew of Assembly B is calculated and the contigs are broken in the critical points (Assembly C). The new contigs are arranged based on the reference genome and treated by jFGap producing assembly D (Fig. 1-step 4). To preserve correctly assembled contigs within Fuzzy GC Skew critical points, contigs of assembly D are compared with those of the Assembly B, recovering the original sequences and obtaining Assembly E (Fig. 1-step 5). The contigs from Assembly E are additionally processed by jFGap with an extension of contigs ends from 300 bp to 1000 bp generating the final Assembly F (Fig. 1-step 6). For each step, output files are generated and include reports, contig sequences, GC Skew graphics, Dotplots-like graphics for assemblies comparisons, list of GC skew critical points and scripts to run QUAST is generated (Fig. 1-step 7).

### Considerations regarding the use of Fuzzy GC Skew algorithm in genome reassembled

**Figure 2:**
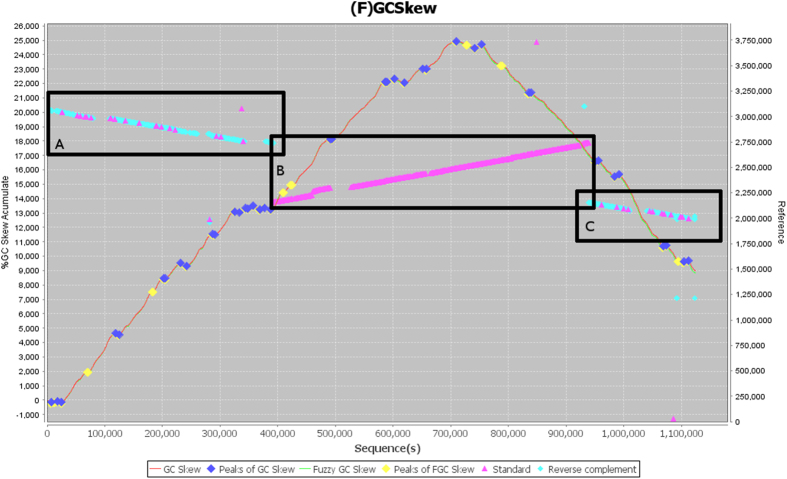
The example of the difference in sensitivity in the detection of critical points in the GC-Skew and Fuzzy-GC-Skew curves. The curves were calculated along a 1.1 Mbp contig in a assembly of *Aeromonas hydrophila*. GC Skew accumulated graphs obtained by classical equation (red line) or by fuzzy method (green line); a 10 kbp window was applied for the calculation. Critical points on GC Skew graphs are shown for classical (blue diamonds) and fuzzy (yellow diamonds) calculations. Regions of divergence in dotplot-like graph (pink and cyan lines) may be cause of variations in the GC Skew curve.

A misassembled contig of 1,100 kbp from *Aeromonas hydrophila* (ctg7180000001875) was chosen to demonstrate the Fuzzy GC skew concept and the determination of the critical points (Fig. 2). Misassembling in the contig is highlighted by Dotplot-like and GC Skew graphs. There are three main regions of high synteny as shown by dotplot-like graph (Fig. 2, boxes A, B, and C), where regions A and C are inverted compared to reference sequence and the region B is in same direction. Many critical points were identified by the algorithm based on GC skew calculated by fuzzy method (Fig. 2, blue and yellow marks).The assemblies are improved when the contigs are broken and realigned these fragments in the reference. This technique to identified misassembly in contigs may be used without references. The standardization of sense of contigs and identification of potential misassemblies based on fuzzy GC Skew are available in graphics mode. In Fig. 2, two cumulative GC skew curves are shown, calculated from different methods: i) using GC skew equation applied in a selected window (blue) and ii) based in a fuzzy equation, applied in the same window (yellow). The observed differences between both methods are justified by the increase in sensitivity provided by Fuzzy version of GC Skew determination.

### Preserving the GC context reduces assembly errors

**Figure 3:**
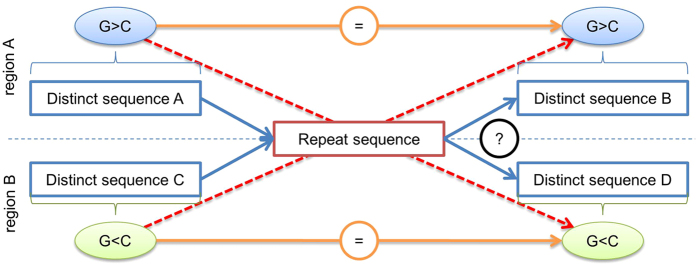
Eulerian path dilemma and GC skew context in genome sequence assembly using de Bruijn graph. The de-Bruijn graph representing a collapsed repeated region (red box) and the path dilemma to choose the path through this region in the assembler. The orange line shows the trend that is not used by assemblers. The red dashed line may be misassemblies.

There are spurious variations in GC Skew observed in misassembled contigs. Our results indicate that assemblers may preserve the sign of the equation GC Skew (G − C)/(G + C) when the dilemma of the path occurs caused by repeat. The Fig. 3 shows a de-Bruijn diagram with the path’s dilemma^19^ of assembler after a repeat region where only the statistical interpretation of the readings coverage.

### The GFinisher validation

**Figure 4:**
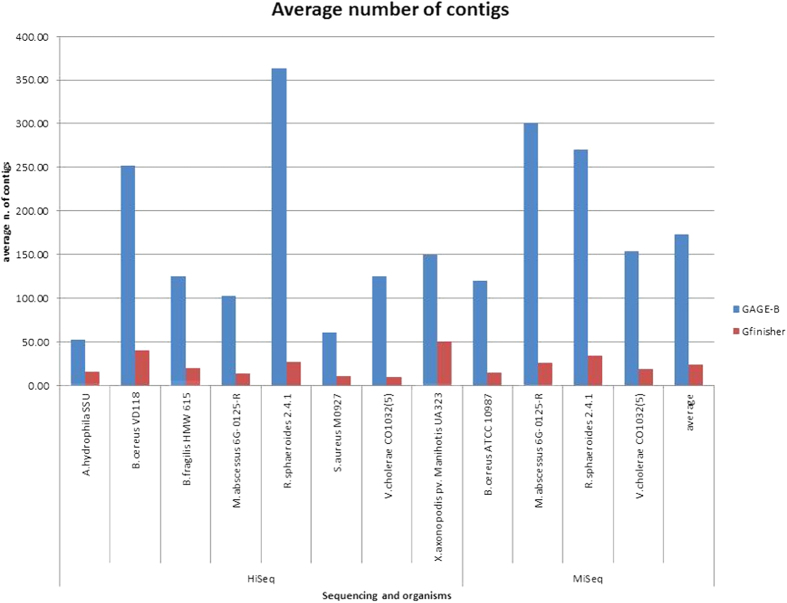
GFinisher improvement in the average number of contigs for 12 prokaryotic genome sequence assemblies available in GAGE-B. Assembled by GAGE-B (blue) and average number of contigs after reassembled by GFinisher (green).

For validation of the proposed methodology, the 96 assemblies provided by GAGE-B were treated by GFinisher (Table S1) and the resulted assemblies were analysed using QUAST. The QUAST reports show significant improvement in metrics for reassembled genome sequences using GFinisher: i) decrease in the average number of contigs by up 86%, from 172.95 to 23.56 (Fig. 4); ii) increase in average size of the N50 from 123,584 bp to 649,701 bp (525%); iii) reduction in the average value of L50, from 27.84 to 4.39 (84%) and; iv) decrease in the average L75 value from 58.61 to 8.53 (85%) – Table S2.

In the GAGE-B study, the MaSuRCA was the most efficient assembler, concerning the number of contigs and N50 metrics, decreasing the number of contigs in 8 assemblies and increasing the N50 in 10 assemblies, out of 12 analysed genomes assemblies^11^. The comparison of average number of contigs reported by GFinisher and GAGE-B is presented in Table 1. Still this table shows the results obtained with the strategy of break the contigs based on GC Skew cumulative, according to these data, the MaSuRCA is the assembler that has the highest average number of contigs chimerical.

### The GFinisher

The GFinisher helped to improve genome sequence assemblies of 8 bacterial genomes with significant decrease in the number of contigs, even leading to bacterial genome sequencing closing. GFinisher is a free, open-source and user-friendly program that can easily be integrated to other pipelines. It uses biological patterns information to the assembly process to reduce complexity and improve the performance of assemblies outputted by many assembler programs. Fuzzy GC skew has a high processing cost, but can improve accuracy in the location of the critical points of the curve GC skew. This allows the detection of misassembled regions and its correction by GFinisher. The assembly programs that adopt the strategy of maintaining the G > C or G < C to solve the Eulerian path dilemma in de-Bruijn graphs or in OLC overlaps will probably reduce the number of errors in the assembly of contigs and in the complete genome sequence.

## Discussion

The combination of different assemblies to close bacterial genomes, as attempted in various works, failed and this procedure was dismissed by the GAGE-B work. This failure reflects the fact that the assemblers used are unable to handle the combinatorial explosion provided by the genomic repeated regions. In the present work this problem was addressed by the use of a new concept, applying a Fuzzy method to calculate GC Skew along the contig sequences from an assembly, precisely detecting potential misassembled regions, indicating breaking points in contigs sequences and rearrange them in a more precise manner using a reference genome. The Fuzzy GC Skew approach revealed itself to be highly effective in genome assembly and constituted a fundamental algorithm in GFinisher. Further, several parameter options are allowed to be adjusted in GFinisher for a particular characteristics of organism and/or complexities in genomes, leading to a further refinement of the final assembly. The default parameter values were determined by experimentation, aiming to favour a wider range of application.

The GFinisher is executed from three input files: (1) an assembly of a target genome, (2) a reference genome, and (3) alternative assemblies of a target genome. All additional parameters are informed through the a configuration file.

In a typical utilization of GFinisher, if original reads are available, the user generates many *de novo* assemblies using different assemblers tools and parameters. The assembly with the highest N50 and the lowest L50 can be imputed as target genome assembly in GFinisher and the remaining assemblies as alternative assemblies. The contigs of alternative assemblies are used to anchor regions adjacent to gaps and to close the gaps using jFGap. Our analyses results indicate that assemblies generated by variation in parameter set could help in the final composed assembly. However, assemblies produced from Assemblers using different approaches and algorithms give the best results. For example, a combination of OLC and de-Bruijn algorithms, as part of the closing assembly strategy, is recommended and was used in this work.

The reference genome, that can be a *de novo* draft genome, is used to organize the contigs and solve the problem of the combinations resulting from repeated regions. The use of a reference genome by GFinisher does not necessarily imply that the tool perform an “assembly by reference”, since GFinisher will not use sequence reads to produce contig sequences and it will not perform new alignments of reads with the reference genome. The purpose to use a reference genome is the arrangement of contigs order based on the assumption that organisms with close taxonomic and/or sharing similarity in their sequences conservation in gene order as well as present similar repeated regions and genome arrangements. If this assumption is valid for the target assembly, it is possible that for gaps between contigs, there exists a contig in the alternative assemblies that could anchor the target contigs and close the gap. However, the absence of this specific contig in alternative assemblies may be occur due to low genome coverage, a high G + C region, difficult to sequence, repeat regions, DNA inversions or even regions of lateral gene transfer.

All data used in the GAGE-B project had a reference genome of a closely related species and comparison of these sequences with the complete genomes available at NCBI revealed that it was not possible to evaluate the minimum similarity limit between genome sequences.

In the absence of a closely related complete reference genome the following strategy was used in the assembly of the genome of *Herbaspirillum hiltneri* N3^20^. In this case, initially over 800 contigs of *Herbaspirillum lusitanum* P6-12^21^ were mapped on the complete genome of *Herbaspirillum seropedicae* SmR1^22^ and the obtained aligned scaffolds were used as reference genome to order the contigs of *H. hiltneri* N3.

In cases where the reference genome is taxonomically distant or has low similarity, it is advisable to verify if all contigs of the target assembly are anchored to the reference genome. This information is available in the reports issued by GFinisher.

The window size in the Fuzzy GC skew algorithm is an important parameter. The default size used was 10 kbp and it is inversely proportional to the number of detected critical points. Values significantly above 30 kbp reduce sensitivity and less than 5 kbp cause increased fragmentation of the target assembly.

In summary, we present a new method to finish a genome sequence or reduce number of contigs using alternative assemblies from different assemblers. The method makes use *a priori* information of the genome sequence, namely the GC skew, to suggest point of break and reordering of the contigs based on a reference sequence. The method applied to the GAGE-B data set resulted in a reduction of approximately 86% of the number of contigs and increase of N50 of 525%.

## Methods

### Data source

In this work we analysed the 96 bacterial genome assemblies available from GAGE-B paper. The genome size of the organism varied from 2.9 MB to 5.4 MB and had a GC percentage from 33 and 69. The analysed genomes were from the bacteria *Aeromonas hydrophila* SSU (access number NC 008570), *Bacillus cereus* ATCC 10987 and VD118 (NC 003909, NC 005707), *Bacteroides fragilis*HMW615 (NC 016776), *Mycobacterium abscessus* 6G-0125–R (NC 010394, NC 010397), *Rhodobacter sphaeroides* 2.4.1 (NC 007488, NC 007489, NC 007490, NC 007493, NC 007494, NC 009007, NC 009008), *Staphylococcus aureus* M0927 (NC 010063, NC 010079, NC 012417), *Vibrio cholerae* CO1032 (NC 002505, NC 002506) and *Xanthomonas axonopodis* pv. Manihotis UA323 (NC 016010). The genomic sequences of these organisms were obtained from the NCBI Genbank and used as reference. Two main types of algorithm: (i) the overlap-layout-consensus (OLC) and (ii) algorithms based on a de-Bruijn graph were used in the assemblies carried out by GAGE-B and they are listed in Table S3^23^,^24^,^25^,^26^,^27^,^28^,^29^,^30^. It is worth noting that the MaSuRCA assembler is the only one that uses both algorithms.

### Organizing the contigs based on reference genomes

The new contig set was organised by mapping on its respective reference genome using jContigSort application^31^.

The jContigSort strategy consists in obtaining subsequence of the reference genome corresponding to the specified kmer, which are then indexed in a *HashMap* collection with their locations. The subsequence of each contig are then searched in the *HashMap* collection aiming to identify their locations in the reference genome. These locations are then stored in an array and clustered (kmeans). The cluster containing the largest number of locations or density is selected and its location in the reference genome is identified by the median element of the cluster. This procedure is repeated for all contigs allowing a systemic ordering of the contigs in relation to the reference genome. In GFinisher the default value for the kmer is 15, but the best results can be obtained with odd values between 11 and 31. Lower kmer sizes reduce the number of keys and increase the likelihood of subsequence intersections, but increase computer memory consumption and the clustering processing time. Higher values increase the number of keys and reduce the chance of subsequent intersection. Therefore, the level of similarity of the target draft genome to the reference genome must be considered in deciding which kmer size is chosen.

### Dotplot analysis

We developed a new program to pairwise compare nucleic acid sequences, to align contigs of the draft genome to the reference genome and to present the data as a Dotplot graph. The contigs of the draft genomes are color identified. This stage allows the ordering of the contigs according to the reference genome before the refining of the genome assembly in analysis. This approach allows the evaluation of the improvement of the genome assembly refinement.

### GC Skew and Fuzzy GC Skew

A new algorithm was developed again to determine the GC skew as part of the GFinisher. The GC skew graphs of the reference genomes constructed by this algorithm were identical to those found in the University of Lausanne site http://www2.unil.ch/comparativegenometrics/.

The GC skew are calculated as (G − C)/(G + C) for a window sliding (2d + 1) along the sequence. The window length limits the precision in the location of critical points of curve. Smaller values for fragment lengths produce a larger number of critical points and a larger window value reduces the precision in the identification of critical points. We also developed the Fuzzy GC Skew function to improve precision in the identification of critical points. The Fuzzy GC Skew function was defined by equation 6 and the calculation steps are shown by equations 1, 2, 3, 4, 5: Were S is a DNA sequence of length n


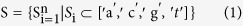


and 2d + 1 is the length of a moving window, where “d” is the length of a stretch of DNA sequence neighbouring both upstream and downstream a specific nucleotide residue and defined by researcher.

The triangular function T is given by:


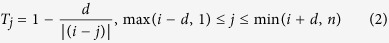


The G and C functions are defined as:


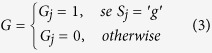



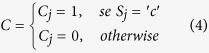


The propose Fuzzy GC skew function U is given by equation 5:


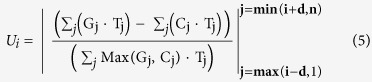


And the accumulated Fuzzy GC Skew A is given by equation 6:


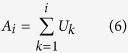


The function fuzzy GC Skew (U, 5 is a moving average of the GC Skew, where each element of U corresponds to a specific nucleotide base of the original sequence S (equation 1). The same property can be seen in the accumulated Fuzzy GC Skew function (equation 6). It is observed, however, that the property is

### Breaking contigs

An algorithm was developed to break down contigs at critical points of the Fuzzy GC Skew curve longer than 1,000 bp. The critical points are the local minimum and local maximum points on the accumulated Fuzzy GC Skew curve. The change in signal observed in calculated difference between two consecutive points allows the identification of critical points.

### jFGap

The FGap^32^ originally written in Matlab was rewritten in Java, preserving the same concept, and improved for working with contigs. This application is part of the GFinisher as jFGap.

### Assemblies’ comparison and evaluation

The original genome assemblies and the results of refining and finishing genomes by GFinisher were evaluated by QUAST. Metrics such as Number of contigs, N50, N75, L50 and L75 were analysed (Table S2). These were similar to those used in comparisons made by GAGE-B.

## Additional Information

**How to cite this article**: Guizelini, D. *et al*. GFinisher: a new strategy to refine and finish bacterial genome assemblies. *Sci. Rep.* **6**, 34963; doi: 10.1038/srep34963 (2016).

## Supplementary Material

Supplementary Information

## Figures and Tables

**Table 1:** Average number of contigs and reduction ratio obtained by GFinisher.

Assembler	Contig/Scaffold	Average number of contigs					Correction Rate
		GAGE-B	GFinisher intermediate assemblies		Reduction rate between the GFinisher and GAGE assemblies (%)		
		(N)	(B)	(F)	Before error detection (N − B)/N	After error correction (N − F)/N	
ABySS	Contig	176,17	40,33	27,67	77,11	84,30	7,19
ABySS	Scaffold	164,00	42,75	28,67	73,93	82,52	8,59
CABOG	Contig	219,08	26,33	18,00	87,98	91,78	3,80
CABOG	Scaffold	187,25	26,42	18,33	85,89	90,21	4,32
MaSuRCA	Contig	102,75	43,33	15,83	57,83	84,59	26,76
MaSuRCA	Scaffold	99,50	43,33	15,83	56,45	84,09	27,64
MIRA	Contig	215,58	69,83	40,58	67,61	81,18	13,57
SGA	Contig	344,92	31,25	25,33	90,94	92,66	1,72
SGA	Scaffold	305,50	26,83	21,27	91,22	93,04	1,82
SOAPdenovo2	Contig	163,42	32,25	24,17	80,27	85,21	4,95
SOAPdenovo2	Scaffold	130,75	32,25	24,17	75,33	81,52	6,18
SPAdes	Contig	91,67	29,08	20,64	68,27	77,49	9,21
SPAdes	Scaffold	79,75	30,08	22,75	62,28	71,47	9,20
Velvet	Contig	201,00	35,75	24,08	82,21	88,02	5,80
Velvet	Scaffold	112,92	35,75	24,08	68,34	78,67	10,33
Average		172,95	36,37	23,43	78,97	86,45	7,48

Columns B and F represent the average number of contigs obtained by GFinisher after the second and last steps described in the flowchart of Fig. 1. The rate of reduction of the average number of contigs between GFinisher and GAGE-B, before and after error detection by Fuzzy GC Skew algorithm, is also shown in the 6^th^ and 7^th^ columns. The last column shows the correction rates.
